# Differential expression of miRNAs in the serum of patients with high-risk oral lesions

**DOI:** 10.1002/cam4.17

**Published:** 2012-07-19

**Authors:** Sara Ann MacLellan, James Lawson, Jonathan Baik, Martial Guillaud, Catherine Fang-Yeu Poh, Cathie Garnis

**Affiliations:** 1 Department of Integrative Oncology, British Columbia Cancer Research CentreVancouver, BC, Canada; 2 Oral Biological and Medical Sciences, University of British ColumbiaVancouver, BC, Canada; 3 Division of Otolaryngology, Department of Surgery, University of British ColumbiaVancouver, BC, Canada

**Keywords:** Biomarkers, circulating microRNAs, oral squamous cell carcinoma

## Abstract

Oral cancer is one of the most commonly diagnosed cancers worldwide. Disease is often diagnosed at later stages, which is associated with a poor 5-year survival rate and a high rate of local recurrence. MicroRNAs (miRNAs), a group of small, noncoding RNAs, can be isolated from blood serum samples and have demonstrated utility as biomarkers in multiple cancer types. The aim of this study was to examine the expression profiles of circulating miRNAs in the serum of patients with high-risk oral lesions (HRLs; oral cancer or carcinoma in situ) and to explore their utility as potential oral cancer biomarkers. Global serum miRNA profiles were generated using quantitative PCR method from 1) patients diagnosed with HRLs and undergoing intent-to-cure surgical treatment (*N* = 30) and 2) a demographically matched, noncancer control group (*N* = 26). We next honed our list of serum miRNAs associated with disease by reducing the effects of interpatient variability; we compared serum miRNA profiles from samples taken both before and after tumor resections (*N* = 10). Based on these analyses, fifteen miRNAs were significantly upregulated and five were significantly downregulated based on presence of disease (minimum fold-change >2 in at least 50% of samples, *P* < 0.05, permutation). Five of these miRNAs (miR-16, let-7b, miR-338-3p, miR-223, and miR-29a) yielded an area under the ROC curve (AUC) >0.8, suggesting utility as noninvasive biomarkers for detection of oral cancer or high-grade lesions. Combining these serum miRNA profiles with other screening techniques could greatly improve the sensitivity in oral cancer detection.

## Introduction

Oral cancer is one of the most commonly diagnosed cancers worldwide, and in high-risk countries (e.g., in South-East Asia) this disease represents ∼25% of all new cancers [1]. Oral cancer carries a poor prognosis and, despite advances in treatment, overall survival rates for patients have seen little improvement. This poor prognosis is partially due to frequent diagnosis of disease at later, less-treatable stages [1]. Novel approaches for earlier detection are needed for more accurate patient stratification and management – and improved outcomes.

MicroRNAs (miRNAs) are small, noncoding RNAs that negatively regulate gene expression by preventing translation of specific mRNA transcripts and are known to play a role in many cancers (including oral) [2, 3]. Recent studies have shown that miRNAs are very stable in serum and plasma samples and are resistant to RNase activity [2]. This stability translates into consistent miRNA expression levels among individuals, making serum miRNAs attractive biomarker candidates. Furthermore, profiling of plasma and serum has demonstrated altered miRNA levels associated with cancer and other disease states [2].

There have been reports of differential miRNA expression in plasma from patients with oral squamous cell carcinoma (OSCC) or esophageal cancer when compared with healthy controls [4–6]. Individual miRNAs examined in these studies were selected a priori, based on direct study of cancer tissues. Other data, however, suggest that miRNAs showing disease-associated expression changes in blood are not necessarily the same ones differentially expressed in cancer tissues; tumor cells may selectively release specific miRNAs via exosomes into the extracellular environment, hence miRNA expression patterns in tumor and serum samples from the same patient might not correlate [7, 8]. Furthermore, plasma miRNA expression has been shown to be more variable (as compared with serum expression) [9]. This inconsistency may be due to contamination of platelet miRNAs in plasma samples [10], a reality that makes serum samples more attractive for blood biomarker analysis.

We evaluated global miRNA expression in serum samples taken from patients with OSCC/oral carcinoma in situ (CIS) and from a group of demographically matched control individuals with no cancer to identify miRNAs with disease-associated serum expression. We further compared global serum miRNA expression profiles in blood samples taken from individuals with disease that were collected before and after surgery, honing our list of disease-associated serum miRNA candidates through internally matched comparison. This work represents the first-ever attempt to delineate serum miRNA biomarkers for head and neck malignancy, and one of the first attempts to define such markers for any epithelial precancer.

## Materials and Methods

### Sample collection

Serum samples were collected from 33 patients with high-risk oral lesions (HRLs, i.e., CIS or OSCC cases) undergoing curative resection treatment between the dates of March 2010 and June 2011. Three of these samples evinced hemolysis (which can alter circulating miRNA levels [10]) and were excluded. Additional samples from 10 of these patients were obtained 6 months after surgery for comparison with presurgery samples. For two of these patients, fresh frozen tissue samples from biopsies of the lesion were also collected.

Serum samples from a noncancer control group (*N* = 26) that were demographically matched to the above cohort for age, sex, smoking status, and ethnicity were also collected (Table 1). These samples were drawn from an ongoing pan-Canadian lung cancer screening study and were collected between the dates of December 2008 and March 2011. All blood samples were collected in SST vacutainer tubes and kept at room temperature (30 min) to allow clotting. Clotted samples were centrifuged at room temperature (15 min, 1500 rcf), aliquoted, and frozen at −80°C (within 2 h).

**Table 1:** Characteristics of the study cohort

	CIS	OSCC	Noncancer controls
Total patients	14	16	26
Mean age	64	62	62
Age range	50–84	51–93	50–75
Number of males	12	9	13
Number of females	2	7	13
Former smokers	8	10	14
Current smokers	5	3	12
Nonsmokers	1	3	0
Mean smoker pack- years	24	30	43
Pack-year range	0–60	0–156	30–80
Ethnicity			
Caucasian	13	14	26
Other	1	2	0

### RNA purification

Total RNA was extracted from 200 *μ*L of serum using miRNeasy Mini Kit (Qiagen, Toronto, ON, Canada) according to manufacturer instructions, except that 1.25 *μ*L of MS2 carrier RNA (Roche Applied Science, Laval, QC, Canada) per 200 *μ*L of serum was added to the QIAzol Lysis Reagent prior to RNA purification. Purified RNA was resuspended in 50 μL of nuclease-free water and stored at −80°C prior to assaying miRNA expression. For freshly frozen disease tissues, RNA was purified via standard TRIzol (Life Technologies Inc., Burlington, ON, Canada) protocol after microdissection by an oral pathologist (CFP).

### miRNA quantification by qRT-PCR

Due to low RNA yields in serum, the concentration of purified RNA could not be reliably measured; hence, fixed volumes of eluted RNA (19.2 *μ*L for 768 reactions) were used for miRNA expression assays as previously reported [4, 6, 11]. RNA was reverse transcribed using the miRCURY LNA Universal RT miRNA PCR, Polyadenylation and cDNA synthesis kit (Exiqon Inc., Woburn, MA). cDNA was then quantified by quantitative real-time PCR (qRT-PCR) on the miRNA Ready-to-Use PCR, Human panel I and panel II with the miRCURY LNA Universal RT miRNA PCR, SYBR Green master mix according to the manufacturer's protocol (Exiqon Inc.). C_p_-values and ROX reference dye normalization were calculated using Viia7 Software (Life Technologies Inc.). All assays were inspected for distinct melting curves – those with >1 T_m_ and those detected within 5 C_p_'s of the negative control (C_p_ > 35) were excluded from analysis.

### Data analysis

qRT-PCR results were exported to GenEx (MultiD Analyses AB, Goteborg, Sweden) and normalized using interplate calibrators on the miRNA Ready-to-Use panels. The mean expression value of all expressed miRNAs in a given sample was used for further normalization [12]. To compare the matched pre- and postsurgery samples, fold-change analysis was used (2^(presurgeryΔCp − postsurgeryΔCp)^).

Analysis of variance (ANOVA) followed by Tukey HSD post hoc testing (calculated using Statistica [Statsoft, Tulsa, OK]) was used to compare serum miRNA profiles between CIS, OSCC, and noncancer control cases. A permutation test, conducted using miRNAs that were present at detectable levels in ≥60% of samples, was used to identify miRNAs significantly deregulated between disease and control cases [13]. All *P*-values were corrected using the Benjamini and Hochberg method for multiple testing. Average-linkage hierarchical clustering was calculated and displayed using Genesis software (http://www.genome.tugraz.at). Receiver operating characteristic (ROC) curves and area under the curve (AUC) plots were generated using the ROCR package in R [14].

## Results

We assayed miRNA expression in serum from 56 subjects (30 HRL patients and 26 noncancer controls). Of 742 miRNAs interrogated by qRT-PCR, 565 were detected in ≥1 of our samples and 58 were detected in every sample. In addition, 502 miRNAs were detected in ≥1 HRL sample and 69 miRNAs were detected in all HRL samples.

The HRL cohort consisted of 14 CIS and 16 OSCC cases. As miRNA expression can vary based on histopathological stage [15], we first asked whether these two groups had distinct serum miRNA profiles compared to controls. A one-way ANOVA of all 56 samples showed significant differential expression of six miRNAs that were specific to CIS versus control comparisons. Two miRNAs were found to have significantly different expression between CIS versus control and CIS versus OSCC (though not different between control and OSCC). Also, ten miRNAs were found to have differential expression between control and both CIS and OSCC, but not between CIS and OSCC. And, four miRNAs showed a difference specific to the OSCC group when compared with control (Table S1). Ultimately, as CIS has a high chance of progressing to OSCC, we treated CIS and OSCC as a single HRL cohort. A permutation test comparing HRL samples to the demographically matched control samples was conducted on all 56 cases using the global-mean normalized data (ΔC_p_). A total of 45 miRNAs were significantly upregulated (*P* < 0.001) and ten miRNAs were significantly downregulated in the HRL samples compared with controls.

To ensure that the observed differential miRNA expression between the HRL and control groups was due to the presence of an oral lesion – and to avoid interpatient variability – we next compared miRNA profiles for serum samples taken from matched pre- and postsurgical serum samples (*N* = 10). Serum samples contributing to this analysis were taken immediately prior to surgery and 6 months following surgical resection. An average-linkage hierarchical clustering analysis on the 50 miRNAs with the highest standard deviation showed that the serum miRNA profiles clustered according to the presence/absence of disease – and not according to the source patient (Fig. S1).

**Figure 1:**
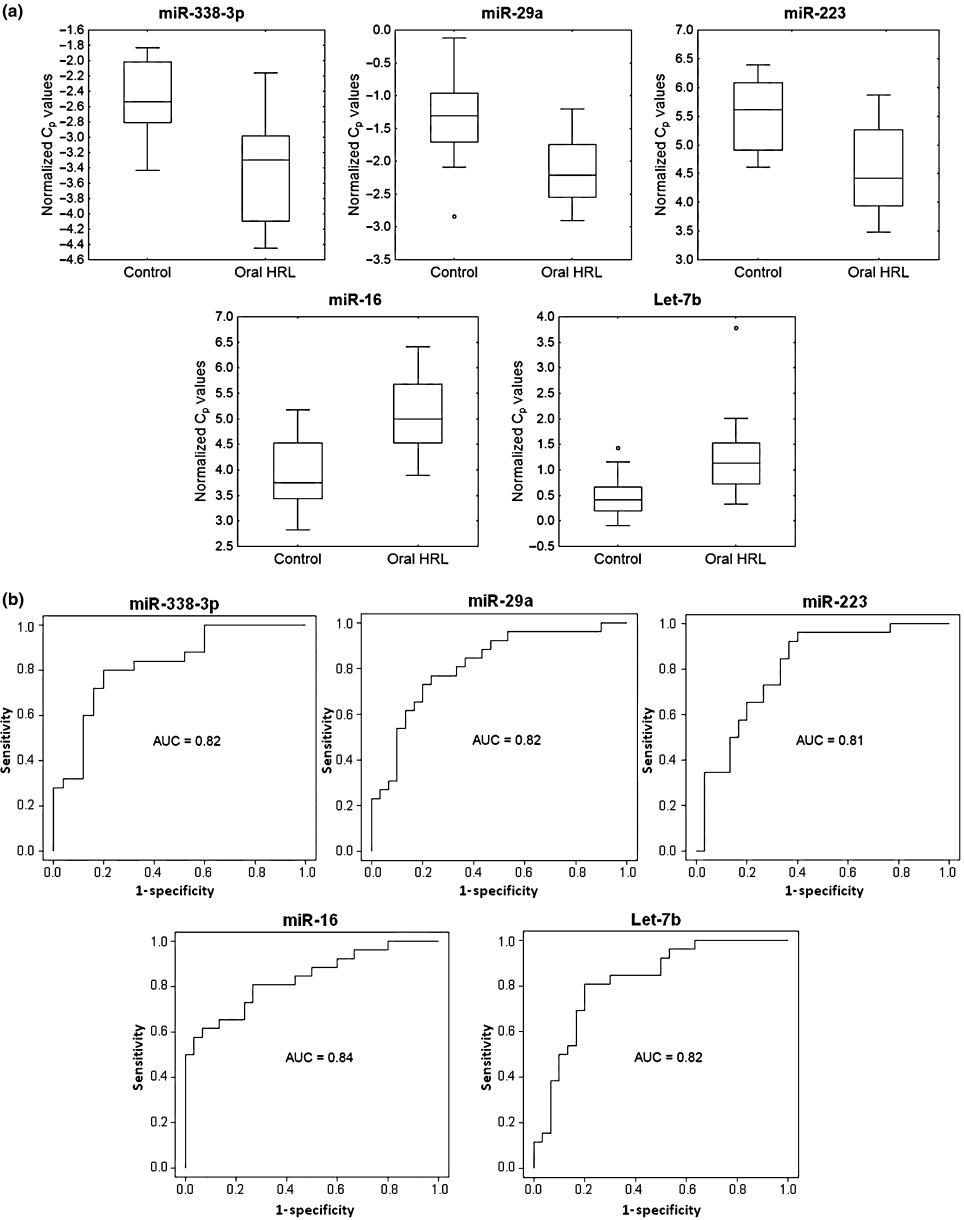
(a) Expression levels of the five candidate miRNAs in the serum of oral HRLs (*N* = 30) and noncancer control (*N* = 26) patients. Scale at *y*-axis represents C_p_ values normalized to the global mean. Line inside the box: median, box: interval between the 25th and 75th percentiles, whiskers: interval between the 10th and 90th percentiles, circles: outliers. (b) ROC curve analysis conducted on the cancer versus control cases showing the five most significantly deregulated miRNAs. miR-333-3p yielded an AUC of 0.82 (95% CI: 0.71–0.94) with 80.0% specificity and 80.0% sensitivity in identifying oral HRLs, miR-29a yielded an AUC of 0.82 (95% CI: 0.70–0.93) with 76.7% specificity and 76.9% sensitivity, miR-223 yielded an AUC of 0.81 (95% CI: 0.69–0.92) with 60.0% specificity and 96.2% sensitivity, miR-16 yielded an AUC of 0.84 (95% CI: 0.73–0.94) with 93.3% specificity and 61.5% sensitivity, and let-7b yielded an AUC of 0.82 (95% CI: 0.71–0.93) with 80.0% specificity and 80.8% sensitivity.

A fold-change analysis of miRNA expression levels showed upregulation of 32 miRNAs and downregulation of 41 miRNAs by ≥2-fold in ≥50% of presurgical cases (vs. postsurgical ones). Comparing the results from both above analyses – (1) analysis of HRL versus control samples and (2) analysis of pre- versus postsurgical samples – uncovered 15 miRNAs with expression that was significantly upregulated with disease (i.e., cancer patients in comparison #1 and presurgical patients in comparison #2) (Table 2). Five miRNAs were also found to be significantly downregulated with disease in this comparison (Table 2). Follow-up with ROC curve analysis of these 20 candidates identified five miRNAs with AUC values >0.8. Two of five were significantly upregulated in HRL cases compared with controls (miR-16 and let-7b) and three were downregulated in this same comparison (miR-338-3p, miR-223, and miR-29a) (Fig. 1).

**Table 2:** Significantly differentially expressed miRNAs in both cancer versus control cases and pre- versus postsurgery cases

miRNA	*P*-value^a^	% of samples fold-change >2^b^
Upregulated in cancer cases		
miR-16	<1 × 10^−10^	80
let-7b	<1 × 10^−10^	70
miR-26a	1.25 × 10^−10^	60
miR-17	1.37 × 10^−10^	50
miR-19a	2.14 × 10^−10^	80
miR-486-5p	9.15 × 10^−10^	50
miR-92a	7.37 × 10^−9^	60
miR-30e	1.80 × 10^−7^	50
miR-320b	9.97 × 10^−7^	50
miR-451	4.86 × 10^−6^	50
miR-7	1.13 × 10^−5^	60
miR-25	1.55 × 10^−5^	50
let-7a	6.51 × 10^−5^	50
miR-195	1.12 × 10^−4^	50
miR-624^*^	1.33 × 10^−4^	60
Downregulated in cancer cases		
miR-29a	6.67 × 10^−7^	60
miR-223	1.07 × 10^−6^	60
miR-338-3p	1.1 × 10^−6^	80
miR-142-5p	1.69 × 10^−6^	70
let-7d^*^	7.68 × 10^−6^	70

a Determined using a permutation test on cancer versus control samples and corrected for multiple testing using the Benjamini–Hochberg method.

b Calculated with the formula 2^(presurgeryΔCp − postsurgeryΔCp)^ using matched pre- and postsurgery samples.

We also sought to determine whether serum profiles were reflective of tissue miRNA expression in two cases where matched HRL tissue specimens were available. For one patient, 30 miRNAs were upregulated in both tissue and serum, no miRNAs were downregulated in both serum and tissue, and 322 miRNAs were differentially expressed between tissue and serum. For the second patient, 39 miRNAs were upregulated and three were downregulated in both samples, whereas 346 were differentially expressed.

## Discussion

Novel biomarkers detecting oral cancer at early stages could be used to more effectively stratify patients and ultimately improve disease outcomes. By evaluating expression of circulating miRNAs in sera from patients with oral cancer, we identified 20 miRNAs that are significantly deregulated with oral HRLs. This work represents the first-ever global analysis of serum miRNA expression profiles in patients with any head and neck cancer or precancer.

Since the discovery of stable miRNAs in human serum and plasma [2, 16], the utility of circulating miRNAs as biomarkers for diagnosis and management of cancer has been increasingly investigated [17, 18]. Serum-expressed miRNAs have great potential as biomarkers: miRNAs are understood to be key molecular drivers affecting cancer phenotypes (thus making them worthy of direct monitoring) [19] and serum samples for miRNA expression are both cheaply and easily (i.e., noninvasively) obtained, making them feasible to apply in screening populations.

By comparing the serum miRNA profiles of patients with oral cancer to profiles from noncancer controls, we identified 55 miRNAs that were significantly deregulated with disease, demonstrating that the presence of oral HRLs can significantly alter circulating miRNA expression. Six miRNAs were specifically deregulated in the serum of patients with precancerous lesions whereas four miRNAs were specifically deregulated in serum from OSCC patients (Table S1). These stage-specific miRNA profiles may represent alterations in exosomally secreted miRNAs during cancer progression. The presence of specific serum miRNAs associated with precancerous lesions also suggests utility for such miRNAs in the early detection of oral cancer; these easily tested blood markers could be applied as an adjunct to direct investigation of the oral cavity to improve disease detection.

An analysis of serum miRNA levels in pre- and postoperative samples demonstrated that many circulating miRNAs are tumor specific as the levels correspondently decrease or increase after tumor resection (Fig. S1). Because miRNAs are involved in many cell processes [3], the use of these patient-matched samples allowed us to remove interpatient variability. These results suggest that serum miRNAs could also have utility for monitoring disease recurrence, a clinical reality that contributes significantly to continuing poor oral cancer outcomes [1]. Moreover, because treatment (surgery, radiation) can alter the appearance of normal tissue surrounding a lesion, which can delay recurrence detection, serum miRNAs may be applicable as an alternative means of posttreatment monitoring.

Cross-comparison of results from the two above analyses identified 20 serum miRNAs consistently deregulated based on disease status. These miRNAs did not overlap with candidates defined by other oral cancer analyses [4, 5, 20]. One explanation for this could be the use of serum as the miRNA source instead of plasma, as recent reports have shown differential miRNA expression in patient-matched serum and plasma samples [9, 10]. Another significant result was the detection of serum miRNAs upregulated in normal and postsurgery patients as compared to disease/matched presurgical samples (five of 20 miRNAs referenced above). The finding of serum miRNAs upregulated in disease/presurgical samples relative to their comparators is intuitive, as the given HRL still harbored by the patient could be the overexpressed serum miRNA source; absence or removal of this miRNA source would be associated with reduced expression. However, the finding of disease-associated serum miRNAs exhibiting reduced expression relative to nondisease/posttreatment states cannot be explained by this mechanism, suggesting that expression of disease-associated miRNAs in serum may be a product of factors beyond secretion of these miRNAs from diseased cells. Similar results have been reported in other cancer types [6, 9].

ROC analysis of the 20 miRNA candidates identified five miRNAs (miR-16, let-7b, miR-338-3p, miR-223, and miR-29a) that have an AUC >0.8, suggesting that these miRNAs could have utility as biomarkers for oral cancer detection. The two miRNAs that were highly upregulated in sera from patients with oral HRLs (miR-16 and let-7b) have been reported to act as tumor suppressors (downregulating oncogenes like RAS, BCL2) and are known to be downregulated in many cancer types [21–23]. Our result may be a product of selective exosome-mediated release of let-7 miRNA family members into the extracellular environment, with this exclusion serving to preserve cancer phenotypes [7]. Indeed, selective release of tumor-suppressive miRNAs by cancer cells presents a possible explanation for the upregulation of let-7b and miR-16 in the serum of patients with OSCC/CIS. It may also explain the relative lack of correlation between our matched tissue and serum miRNA profiles reported by other groups [8]. Interestingly, miR-16 has been used by a number of studies as an endogenous control to normalize qRT-PCR data from both OSCC tissue and plasma samples [4, 20]; however, our results show that it is the most significantly upregulated miRNA in the serum of patients with oral cancer (suggesting it may not be a reliable endogenous control). The three miRNAs that were highly downregulated in serum from patients with oral HRLs – miR-338-3p, miR-223, and miR-29a – have been shown to be downregulated in esophageal squamous cell carcinoma tissue, OSCC cell lines, and hypopharyngeal squamous cell carcinoma tissue, respectively [24–26]. As mentioned above, the process by which disease-associated downregulation of serum miRNAs is an outstanding issue requiring further investigation.

We report the first-ever analysis of global miRNA expression in serum samples from patients with oral cancer or precancer. By incorporating matched, pre-/postsurgical serum samples into our combined analysis, we have reduced the impact of interpatient variability on our findings. Due to the relatively small number of samples used in this study, further validation in a larger cohort is needed to fully assess the utility of candidate miRNAs as oral cancer biomarkers. We are currently drawing samples from a large clinical trial to facilitate this validation. Given that there is no means available to delineate disease progression likelihood for oral lesions at earlier dysplastic stages (not analyzed here), we are also accruing serum samples from patients harboring this disease stage to determine whether our serum miRNA biomarker candidates are applicable to this patient population. Our results provide a strong rationale for wider evaluation of serum miRNAs as biomarkers for management of oral malignancy. They also suggest that novel processes pertaining to the relationship between disease and serum miRNA expression remain to be uncovered.

## Supporting Information

**Figure S1.** An average-linkage hierarchical clustering analysis of 50 miRNAs with the highest standard deviation across all pre- versus postsurgery cases showing that disease state has more influence on serum miRNA profiles than inter-patient variability. The analysis was performed on mean centered, normalized data.

**Table S1.** Results of a one-way ANOVA followed by Tukey's HSD post hoc to determine significantly differentially expressed miRNAs between ctrl (non-cancer control), CIS (carcinoma in situ), and OSCC (oral squamous cell carcinoma) samples.

## References

[1] Warnakulasuriya S (2009). Global epidemiology of oral and oropharyngeal cancer. Oral Oncol.

[2] Mitchell PS, Parkin RK, Kroh EM (2008). Circulating microRNAs as stable blood-based markers for cancer detection. Proc. Nat. Acad. Sci. U.S.A.

[3] Bartel DP (2004). MicroRNAs: genomics, biogenesis, mechanism, and function. Cell.

[4] Liu CJ, Kao SY, Tu HF, Tsai MM, Chang KW, Lin SC (2010). Increase of microRNA miR-31 level in plasma could be a potential marker of oral cancer. Oral Dis.

[5] Lin SC, Liu CJ, Lin JA, Chiang WF, Hung PS, Chang KW (2010). miR-24 up-regulation in oral carcinoma: positive association from clinical and in vitro analysis. Oral Oncol.

[6] Komatsu S, Ichikawa D, Takeshita H (2011). Circulating microRNAs in plasma of patients with oesophageal squamous cell carcinoma. Br. J. Cancer.

[7] Ohshima K, Inoue K, Fujiwara A (2010). Let-7 microRNA family is selectively secreted into the extracellular environment via exosomes in a metastatic gastric cancer cell line. PLoS One.

[8] Kosaka N, Iguchi H, Yoshioka Y, Hagiwara K, Takeshita F, Ochiya T (2012). Competitive interactions of cancer cells and normal cells via secretory microRNAs. J. Biol. Chem.

[9] Heegaard NH, Schetter AJ, Welsh JA, Yoneda M, Bowman ED, Harris CC (2012). Circulating micro-RNA expression profiles in early stage nonsmall cell lung cancer. Int. J. Cancer.

[10] McDonald JS, Milosevic D, Reddi HV, Grebe SK, Algeciras-Schimnich A (2011). Analysis of circulating microRNA: preanalytical and analytical challenges. Clin. Chem.

[11] Andreasen D, Fog JU, Biggs W (2010). Improved microRNA quantification in total RNA from clinical samples. Methods.

[12] Mestdagh P, Van Vlierberghe P, De Weer A (2009). A novel and universal method for microRNA RT-qPCR data normalization. Genome Biol.

[13] Chari R, Lonergan KM, Pikor LA (2010). A sequence-based approach to identify reference genes for gene expression analysis. BMC Med. Genomics.

[14] Sing T, Sander O, Beerenwinkel N, Lengauer T (2005). ROCR: visualizing classifier performance in R. Bioinformatics.

[15] Cervigne NK, Reis PP, Machado J (2009). Identification of a microRNA signature associated with progression of leukoplakia to oral carcinoma. Hum. Mol. Genet.

[16] Chen X, Ba Y, Ma L (2008). Characterization of microRNAs in serum: a novel class of biomarkers for diagnosis of cancer and other diseases. Cell Res.

[17] Cortez MA, Bueso-Ramos C, Ferdin J, Lopez-Berestein G, Sood AK, Calin GA (2011). MicroRNAs in body fluids – the mix of hormones and biomarkers. Nature reviews. Clin. Oncol.

[18] Kukreja RC, Yin C, Salloum FN (2011). MicroRNAs: new players in cardiac injury and protection. Mol. Pharmacol.

[19] Lu J, Getz G, Miska EA (2005). MicroRNA expression profiles classify human cancers. Nature.

[20] Wong TS, Liu XB, Wong BY, Ng RW, Yuen AP, Wei WI (2008). Mature miR-184 as potential oncogenic microRNA of squamous cell carcinoma of tongue. Clin. Cancer Res.

[21] Takamizawa J, Konishi H, Yanagisawa K (2004). Reduced expression of the let-7 microRNAs in human lung cancers in association with shortened postoperative survival. Cancer Res.

[22] Johnson SM, Grosshans H, Shingara J (2005). RAS is regulated by the let-7 microRNA family. Cell.

[23] Aqeilan RI, Calin GA, Croce CM (2010). miR-15a and miR-16-1 in cancer: discovery, function and future perspectives. Cell Death Differ.

[24] Lin RJ, Xiao DW, Liao LD (2012). MiR-142-3p as a potential prognostic biomarker for esophageal squamous cell carcinoma. J. Surg. Oncol.

[25] Kozaki K, Imoto I, Mogi S, Omura K, Inazawa J (2008). Exploration of tumor-suppressive microRNAs silenced by DNA hypermethylation in oral cancer. Cancer Res.

[26] Kikkawa N, Hanazawa T, Fujimura L (2010). miR-489 is a tumour-suppressive miRNA target PTPN11 in hypopharyngeal squamous cell carcinoma (HSCC). Br. J. Cancer.

