# Supplementary figures and images for: Differential expression of miRNAs in the serum of patients with high-risk oral lesions
Source: Cancer Med. 2012 Jul 19;1(2):268–74. doi: 10.1002/cam4.17 (PMC3544450; doi:10.1002/cam4.17)

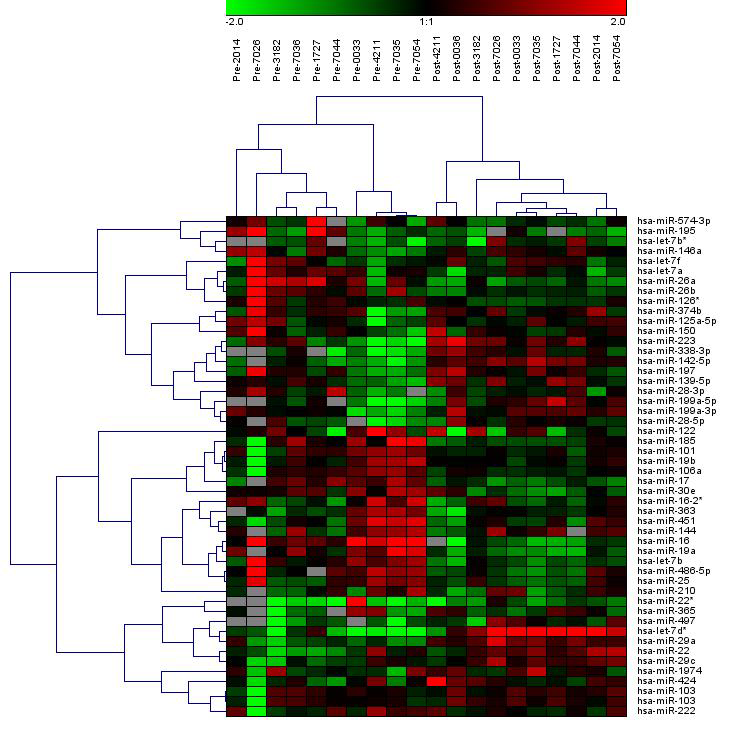

Supplement: Supplementary file 1 [file cam40001-0268-SD1.tif]
