# Supplementary material for: Differential expression of miRNAs in the serum of patients with high-risk oral lesions
Source: Cancer Med. 2012 Jul 19;1(2):268–74. doi: 10.1002/cam4.17 (PMC3544450; doi:10.1002/cam4.17)
Supplement: Supplementary file 2 [file cam40001-0268-SD2.doc]

**Tables**

Table S1. Results of a one-way ANOVA followed by Tukey’s HSD *post hoc* to determine significantly differentially expressed miRNAs between ctrl (non-cancer control), CIS (carcinoma *in situ*), and OSCC (oral squamous cell carcinoma) samples.

| **miRNA** | ***p*-value**^a^ | **Groups that are**  **significantly different** |
| --- | --- | --- |
| miR-197 | 0.0150 | ctrl/CIS |
| miR-125b | 0.0219 | ctrl/CIS |
| miR-342-3p | 0.0282 | ctrl/CIS |
| miR-19a | 0.0331 | ctrl/CIS |
| miR-486-5p | 0.0417 | ctrl/CIS |
| miR-23a | 0.0469 | ctrl/CIS |
| let-7d* | 0.0046 | ctrl/OSCC |
| miR-26b | 0.0300 | ctrl/OSCC |
| miR-26a | 0.0329 | ctrl/OSCC |
| miR-29c | 0.0373 | ctrl/OSCC |
| miR-320a | 0.0177 | ctrl/CIS , ctrl/OSCC |
| miR-142-5p | 0.0040 | ctrl/CIS, ctrl/OSCC |
| miR-16 | 0.0042 | ctrl/CIS, ctrl/OSCC |
| miR-29a | 0.0066 | ctrl/CIS, ctrl/OSCC |
| miR-338-3p | 0.0081 | ctrl/CIS, ctrl/OSCC |
| let-7b | 0.0093 | ctrl/CIS, ctrl/OSCC |
| miR-17 | 0.0367 | ctrl/CIS, ctrl/OSCC |
| miR-150 | 0.0373 | ctrl/CIS, ctrl/OSCC |
| miR-18b | 0.0373 | ctrl/CIS, ctrl/OSCC |
| miR-181a | 0.0480 | ctrl/CIS, ctrl/OSCC |
| miR-223 | 0.0002 | ctrl/CIS, CIS/OSCC |
| miR-423-3p | 0.0026 | ctrl/CIS, CIS/OSCC |

^a^Corrected for multiple testing using the Benjamini-Hochberg method.
